# Reduced benefit from long-term item frequency contributes to short-term memory deficits in dyslexia

**DOI:** 10.3758/s13421-024-01601-z

**Published:** 2024-07-02

**Authors:** Eva Kimel, Luba Daikhin, Hilla Jakoby, Merav Ahissar

**Affiliations:** 1 The Edmond and Lily Safra Center for Brain Sciences, Jerusalem, Israel; 2 Department of Psychology, The Hebrew University of Jerusalem, Edmond J. Safra Campus, Jerusalem, Israel; 3 Present Address: Department of Psychology, The University of York, York, North Yorkshire YO10 5DD UK; 4 Present Address: Department of Communication Disorders, Hadassah Academic College, Jerusalem, Israel

**Keywords:** Short-term memory, Long-term memory, Dyslexia, Item frequency, Individual differences

## Abstract

Dyslexia, a specific difficulty in acquiring proficient reading, is also characterized by reduced short-term memory (STM) capacity. Extensive research indicates that individuals with developmental dyslexia (IDDs) benefit less from exposure, and this hampers their long-term knowledge accumulation. It is well established that long-term knowledge has a great effect on performance in STM tasks, and thus IDDs’ reduced benefit of exposure could potentially reduce their relative performance in such tasks, especially when frequent items, such as digit-words, are used. In this study we used a standard, widely used, STM assessment: the Digit Span subtest from the Wechsler Adult Intelligence Scale. The task was conducted twice: in native language and in second language. As exposure to native language is greater than exposure to second language, we predicted that IDDs’ performance in the task administered in native language will reveal a larger group difference as compared to second language, due to IDDs’ reduced benefit of item frequency. The prediction was confirmed, in line with the hypothesis that reduced STM in dyslexia to a large extent reflects reduced benefits from long-term item frequency and not a reduced STM per se.

## Introduction

Developmental dyslexia is a specific difficulty with literacy acquisition, in spite of adequate hearing levels, normal intelligence, and adequate educational opportunities (*Diagnostic and Statistical Manual of Mental Disorders (DSM-5)*, 2013; Lyon et al., 2003). Dyslexia is usually diagnosed during early school years, and it is a permanent condition (Bazen et al., 2020). The deficit in dyslexia is manifested in lower reading accuracy and speed, impaired word recognition and decoding, and reduced spelling abilities. Another common characteristic of individuals with dyslexia (IDDs) is their poor short-term memory (STM; e.g., Jeffries & Everatt 2004; Melby-Lervåg et al., 2012; Roodenrys & Stokes 2001; Snowling 1981; Snowling et al. 1986).

STM in the general population is often assessed using the Digit Span Task (DST), which is part of the Wechsler Intelligence Scale (Wechsler, 1997). Performance in the DST is correlated with reading fluency, and a functional relation between DST scores and reading skills has been suggested (Jacobson et al., 2011; Jeffries & Everatt, 2004; Naveh-Benjamin & Ayres, 1986; Nicolson, 1981; Roodenrys & Stokes, 2001; Snowling et al., 1986; Snowling, 1981). This implies that individuals with reduced reading skills are expected to have a reduced performance in the DST, and indeed, a reduced performance in the DST has been consistently reported for both adults and children with dyslexia (Gabay et al., 2012, 2015; Gabay & Holt, 2015; Hatcher et al., 2002; Henderson & Warmington, 2017; Howes et al., 1999; Jeffries & Everatt, 2004; Nelson & Warrington, 1980; Ramus & Szenkovits, 2008; Witton et al., 1998; though see Wimmer 1993).

Historically, the poor performance of individuals with IDDs in STM tasks has been linked to deficient phonological representations (e.g., Snowling, 2000) or difficulties in accessing them (Ramus, 2014; Ramus & Szenkovits, 2008). However, recent research has challenged the notion that dyslexia's primary deficit is rooted in poor phonology per se (Nittrouer et al., 2018) as studies conducted in the past 30 years have revealed that individuals with IDDs also suffer from non-phonological deficits and can perform adequately in some tasks that require adequate phonological representations (discussed in Ramus & Ahissar, 2012).

Alternative theories have suggested that abnormal learning processes underlie dyslexia. For instance, the anchoring deficit hypothesis (Ahissar, 2007; Ahissar et al., 2006; Oganian & Ahissar, 2012), which focuses on IDDs' learning patterns, suggests that their ability to benefit automatically from input statistics is impaired. This, in turn, could lead to underdeveloped cortical representations of highly frequent categories (Banai & Ahissar, 2017; Perrachione et al., 2016). Reduced benefits from input statistics among IDDs were reported for exposure to stimuli in the lab (Jaffe-Dax et al., 2015; Lieder et al., 2019), for exposure to native language (Kimel & Ahissar, 2019; Perrachione et al., 2011; Schiff et al., 2016; Schiff & Raveh, 2007; Schiff & Ravid, 2007), and for offline consolidation (Ballan et al., 2023; Smith & Henderson, 2016), which could all have a great effect on the build-up of efficiently accessible long-term knowledge.

Long-term knowledge is generally considered to have a big effect on STM (Bartsch & Shepherdson, 2022; Engle et al., 1992; Jensen & Lisman, 1996; Mayzner & Schoenberg, 1964; Perham et al., 2009; Watkins, 1977), and it has been consistently found to influence span task performance: Scores for words are higher than scores for non-words (Hulme et al., 1991), scores for frequent words are higher than scores for infrequent ones (Hulme et al., 1997; Roodenrys et al., 2002), the accuracy for repeating multi-syllabic non-words is higher when the first syllable has a high frequency (Tremblay et al., 2016), and recall of syllable sequences is better for frequently occurring syllables found in polysyllabic English words compared to less frequent ones (Nimmo & Roodenrys, 2002).

**Fig. 1:**
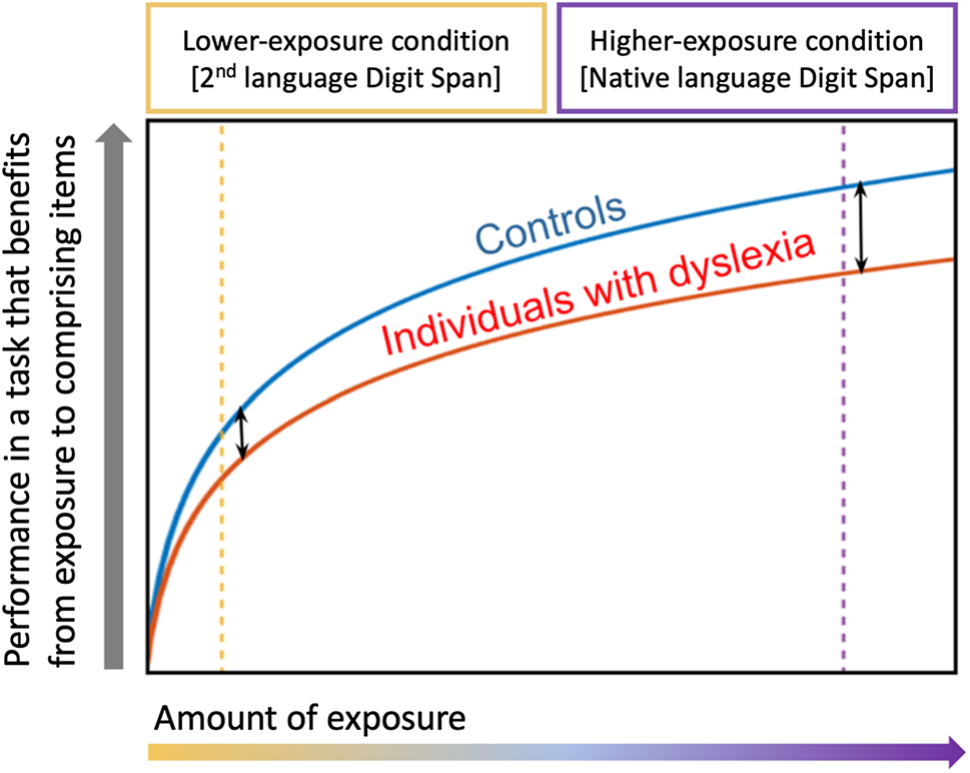
An illustration of the performance in the Digit Span task as an example of a task that benefits from exposure to its comprising items. Performance increases with every exposure, and thus it is better in the high-exposure vs. low-exposure condition. Individuals with developmental dyslexia’s (IDD) performance is reduced as compared to that of non-IDDs, and although their absolute performance improves, their relative difficulty increases with exposure. Adapted with permission from Kimel et al. (2022), made available by the Creative Commons Attribution 4.0 International License.

If IDDs’ benefit of input statistics is reduced, their relative difficulty as compared to peers without dyslexia will become more prominent with more exposure/higher item frequency (illustrated in Fig. 1). Reduced benefit of exposure can significantly hamper IDDs’ performance in the widely used DST since digit-words are highly frequent. This suggestion gains further support from recent studies that emphasize the special effect of everyday long-term exposure to digit-words on performance in the DST (Jones & Macken, 2015, 2018; Majerus et al., 2012), beyond the well-documented effect of item frequency on performance in span tasks in general.

In this study we test the novel hypothesis that the numerous reports on IDDs’ poor performance in DST stem, to a large extent, from reduced benefit of exposure. To test this while controlling for semantics, we compare DST performance in native language versus in second language. Although the second language that we used is highly familiar to all our participants, their exposure to their native language is naturally more extensive, resulting in significantly greater exposure to digit-words in their native language as compared to their second language. We suggest that the benefit from each exposure is lower for IDDs as compared to non-IDDs, therefore we expect that the benefit of item frequency will be smaller among IDDs, resulting in a bigger group difference for the native language condition (more exposure; right-hand side in Fig. 1) as compared to the second language condition (less exposure; left-hand side in Fig. 1).

This prediction might seem counterintuitive, as IDDs are known to have second-language difficulties (Crombie, 1997; Ganschow et al., 1995; Sparks & Ganschow, 1991; Spolsky, 1989). We do not dispute it is difficult for IDDs to acquire a foreign language; however, we propose that despite this difficulty, in a well-controlled experiment, group difference is expected to increase with exposure since IDDs’ benefit from each exposure is smaller, as illustrated in Fig. 1. This also suggests that controls’ spans in native and second language will not be strongly correlated since the contribution of the familiarity-dependent mechanism is much larger in the native language than in the second language. By contrast, in dyslexia, where the unique contribution of native language familiarity is expected to be smaller, we expect a stronger correlation between spans in native and second language. In summary, taking into account the learning dynamics of IDDs and non-IDDs, and the effect of item frequency on STM tasks, we predict a bigger group difference in the DST in native language versus second language (right-hand side vs. left-hand side in Fig. 1).

## Method

The Hebrew University's ethics Review Board, termed The Institutional Committee for the Use of Human Subjects in Research, specifically approved this study.

The native language of both IDDs and controls was Hebrew, and all of them did their schooling in Israel, in Hebrew. Yet, they were all highly familiar with English since English is taught in Israeli schools starting from the third grade, and is widely used. Additionally, the two groups were matched for age and general intelligence. Based on this, we assumed a similar exposure to spoken English and Hebrew among the two groups. This matching allowed us to use the DST in Hebrew as our frequent condition, and the DST in English as an infrequent (though familiar) condition, without introducing semantic differences between the conditions. To validate that the DST scores obtained by Hebrew-speaking participants in English are not saturated, we included an additional group of participants, native English speakers, who performed the DST in their native language.

### Assessment battery

Cognitive assessments:

1. The Block Design task (a subtest from the Hebrew version of the Wechsler Adult Intelligence Scale, WAIS-III; Wechsler 1997) was used as a measure of non-verbal intelligence. This task is often used to match groups for non-verbal reasoning.
2. The Digit Span task (Wechsler, 1997), a standard STM measure.

Reading and phonological measures:

1. Single word reading: unrelated single words, pseudo-words, and non-words. All lists used pointed script to comprise the full information needed for correct pronunciation. The pseudo-words had the morphological structure of real words (Deutsch & Bentin, 1996), whereas the non-words did not follow Hebrew morphology or phonotactics (Oganian & Ahissar, 2012). Participants were instructed to read as accurately and fast as they could.
2. Paragraph reading (Ben-Yehudah et al., 2001). Participants were instructed to read as fast as they could without harming comprehension.
3. Phonological awareness: Twenty word pairs were orally presented (Ben-Yehudah et al., 2001; Ben-Yehudah & Ahissar, 2004). For each pair, participants were asked to swap the initial phonemes of the two words (the "spoonerism" task). If the swap was performed correctly, participants got 1 point, thus the maximum number of points for this task was 20. Participants were instructed to perform the task as accurately and as fast as they could.

### Participants

Participants were recruited around the Hebrew University campuses and at two other colleges in Jerusalem. A power analysis with two-tailed α of .05, power of .8, and a conservative assumption of an effect size of .8 for the group difference for DST in native language (Jeffries & Everatt, 2004) yielded a required sample size of 26 participants per-group (Kohn & Senyak, 2024). A total of 37 control participants and 35 participants with dyslexia took part in an introductory session at the lab that included the assessment battery described above. Participants were paid for their participation and took part in a number of studies at the lab (Kimel et al., 2020, 2022).

All participants had completed school in Israel, in Hebrew. Hebrew was all participants’ mother tongue, and most of them had been born in Israel (the remainder were brought to Israel as young children, and in any case did all their schooling in Israel). All participants knew English, since English is taught in Israeli schools as a foreign language starting from the third grade, and is commonly used in the country.

The assignment to dyslexia and control groups was based on formal diagnosis of dyslexia (based on a declaration of the participants with/without dyslexia of having/not having a formal diagnosis). For the dyslexia group, only participants who reported having early difficulties in reading acquisition were invited. Exclusion criteria for both groups included psychiatric medications (other than those for attention deficit; Oganian & Ahissar, 2012), hearing problems, extensive musical background, and below-average cognitive scores (for details, see Kimel & Ahissar, 2019). Participants who reported having dyslexia but had perfect non-word reading in the introductory session were excluded from the study.

Following the initial assessment battery, which included DST in their native Hebrew, participants were invited to the lab to participate in the DST in their second-language English. Thirty-two IDDs and 30 controls participated (Table 1).

**Table 1:** Participant characteristics

Measure	Controls	IDDs	Group difference (t)
	N = 30 (16 F)	N = 32 (22 F)	
Age (years)	25.8 (2.9)	24.5 (2.9)	n.s.
Block design (scaled)	12.4 (2.8)	12.7 (2.9)	n.s.
Reading accuracy (% correct)			
Single words	97.1 (4.2)	87.8 (7.9)	5.7***
Single pseudo-words	89.7 (11.0)	61.8 (17.7)	7.3***
Single non-words	87.1 (13.2)	52.7 (23.4)	6.9***
Paragraph reading	98.6 (1.4)	94.9 (4.2)	4.6**
Reading rate (words/minute)			
Single words	96.8 (31.9)	68.8 (25.1)	3.8*
Single pseudo-words	58.4 (24.0)	32.1 (10.6)	5.5***
Single non-words	42.1 (15.6)	25.2 (8.7)	5.2***
Paragraph reading	140.4 (23.4)	96.6 (22.3)	7.4***
Phonological awareness: spoonerism			
Accuracy (% correct)	92.2 (6.7)	77.8 (17.2)	4.2**
Rate (items/minute)	10.0 (3.0)	5.6 (3.1)	5.6***

Values are given as means and SDs of the Hebrew-speaking participants with and without dyslexia in reading-related tasks

*IDD* individuals with developmental dyslexia, *F* female participants

*p < .001 ** *p* < .0001 *** *p* ≤ .00001

Participants did not take any ADHD-related medication on the days on which they came to the lab (Oganian & Ahissar, 2012). Eight participants in the dyslexia group reported using ADHD-related medications (e.g., Concerta) on a daily or occasional basis; this subgroup did not significantly differ from the other dyslexia-group participants in age, block design, or their DST Scores in Hebrew or English.

A third group of participants, native English speakers, was recruited, in order to control for native DST scores in English. This group largely overlaps with a group in a previously published study (Kimel et al., 2020). This group was recruited from the Hebrew University’s school of international students, and they were all paid for their participation. These participants’ Hebrew was basic, and they were taking classes in Hebrew as a foreign language. The exclusion criteria applied were similar to those used for the Hebrew speakers. Matching for age and cognitive scores (Block Design) resulted in a group of 29 English-speaking participants (mean (SD) age = 23.6 (5.0) years, block design = 11.6 (2.3)). Adding this group allowed us to verify that span scores in English of the Hebrew-speaking participants are not saturated.

### Procedure

The Hebrew DST was conducted according to the instructions in the Wechsler Adult Intelligence Scale for DST forward (Wechsler, 1997). The experimenter read out sequences of digit-words and were asked to repeat the digit-words in the presented order. The experiment started with two sequences of two digits; sequence length increased by one digit after two sequences of the same length; and the task continued until the participant either failed on two sequences of the same length or reached the maximal sequence length of eight digits (Wechsler, 1997). The performance score was the number of correctly reproduced sequences (a measure frequently used in span tasks; see, e.g., Oganian & Ahissar, 2012). The English-speaking participants did not possess sufficient Hebrew proficiency to take a DST in Hebrew.

The English DST was conducted using a digital recording of a native English speaker. The nine digit-words (one to nine) were recorded separately and then combined into sequences of increasing length. These sequences were played to the participants via headphones. Participants were asked to repeat each sequence immediately after it ended, and their responses were recorded. We asked the Hebrew-speaking participants whether they had translated English digit-words to Hebrew, and they reported that they had not.

## Results

**Fig. 2:**
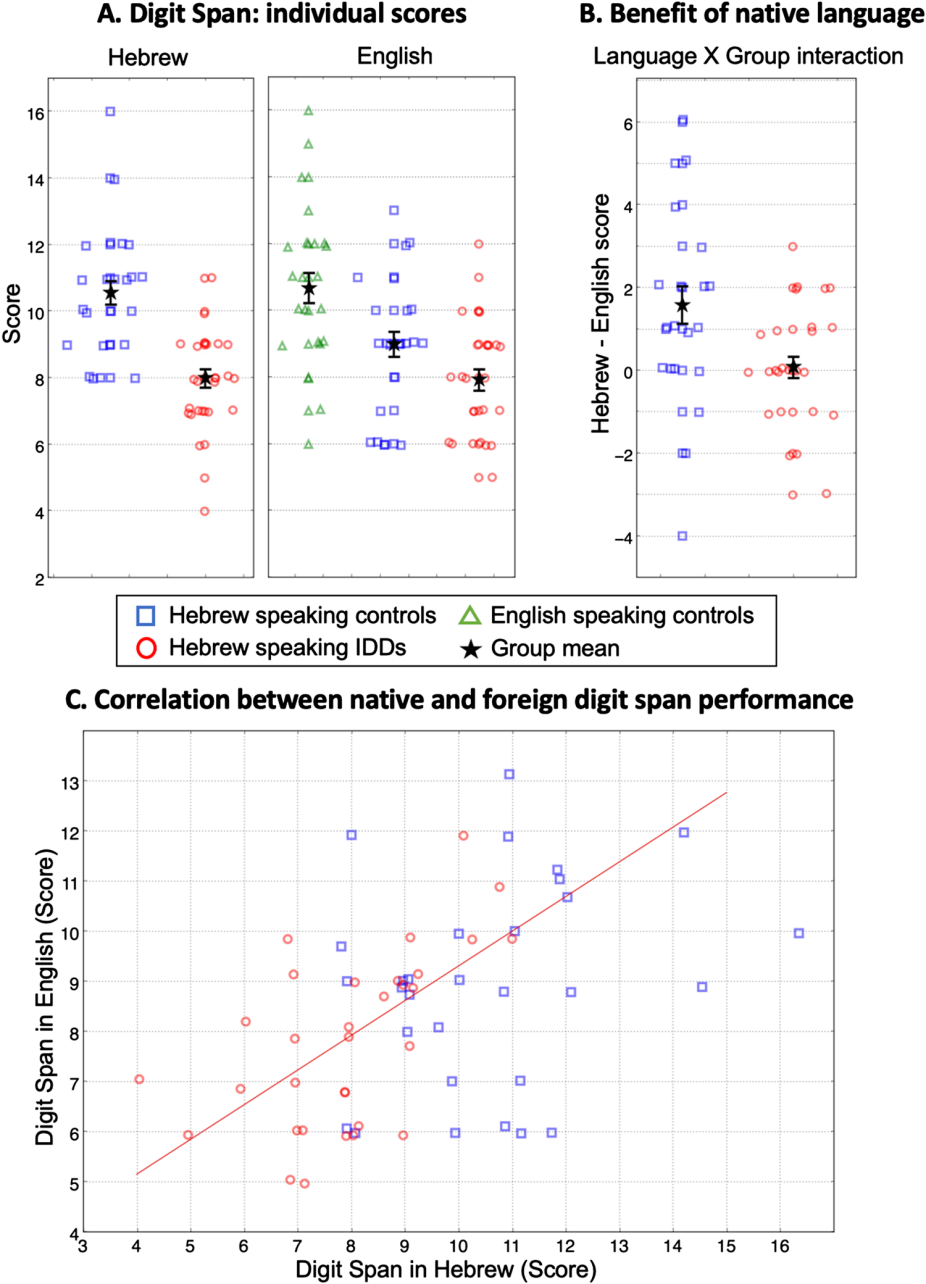
**A**. *Left:* Digit Span Scores of Hebrew-speaking controls (blue squares) and individuals with developmental dyslexia (IDDs) (red circles) tested in Hebrew. *Right:* Scores of English-speaking controls (green triangles), Hebrew-speaking controls (blue squares), and IDDs (red circles) tested in English. **B**. Difference in scores between English and Hebrew, measuring the benefit of frequency/exposure for digit-words. Hebrew-speaking controls benefit more than Hebrew-speaking IDDs from performing the span in Hebrew, their native language. Although Hebrew-speaking IDDs’ raw scores are the same in English and Hebrew, it might still reflect a benefit in Hebrew, in which digit-words are longer than digit-words in English. **C**. A scatter plot of Digit Span scores in English vs. scores in Hebrew. Scores of Hebrew-speaking IDDs are highly correlated (Pearson: r = .60, *p* < .001; red line), whereas those of Hebrew-speaking controls are not (Pearson: r = .25, p = .179). Symbols denote individual scores. Error bars denote one SEM. The values are slightly jittered for display purposes

We first verified our assumption that DST performed in English by Hebrew-speaking participants is not saturated. Namely, we tested whether Hebrew-speaking control participants’ spans in English are smaller than the spans of Native English speakers. To test that, we compared the English and Hebrew speakers’ scores for the English-administered DST. Indeed, Hebrew-speaking controls showed significantly lower scores in English (*t* = 2.89, *p <* .005, Cohen's* d* = .75; Fig. 2A) even though the use of English digit-words is common among Hebrew speakers. This difference established that span scores continue to increase even after massive exposure (Fig. 1).

Importantly﻿, this group difference was significant even though English-speaking controls did not differ in their scaled DST score from the Hebrew-speaking controls (mean (SD) of native language DST: Hebrew-speaking controls = 11.5 (2.9), English-speaking controls = 11.6 (2.5)), although due to English digits being shorter, they were expected to score even higher (Guérard et al., 2012; Guitard et al., 2018; Naveh-Benjamin & Ayres, 1986; Service, 1998).

To test our main prediction that IDDs’ scores would be particularly impaired in high-frequency native-language items (a *Group* × *Language* interaction), we analyzed the number of correctly reproduced sequences in each condition (score) using a mixed-design analysis of variance (ANOVA), with *Language* (native – Hebrew vs. second – English) as a within-participant factor, and *Group* (Hebrew-speaking controls vs. Hebrew-speaking IDDs) as a between-participant factor. Though restricted by the interactions, overall scores of controls were higher than those of IDDs (main effect of *Group*: *F*_(1,60)_ = 21.38, *p <* 10^-4^, *η2 = .*263; Fig. 2A). As expected, scores were also higher for participants’ native language, Hebrew, as compared with those for non-native English (main effect of *Language*: *F*_(1,60)_ = 10.06, *p <* .002, *η2 = .*144; Fig. 2A).

As predicted, performing the DST in participants’ native language benefited controls significantly more than it did IDDs (*Group* × *Language*:* F*_(1,60)_ = 8.57, *p <* .005, *η2 = .*125; controls: *mean difference =* 1.57, *SE = .*37, *p <* 10^-4^, IDDs: *mean difference = .*06, *SE = .*36, *p =* .862), and group difference was smaller in the infrequent (English) condition compared with the frequent (Hebrew) condition (native (Hebrew) language: *F*_(1,60)_ = 32.77, *p <* 10^-6^, *η2 = .*353, Cohen's *d =* 1.38, second (English) language: *F*_(1,60)_ = 4.71, *p <* .034, *η2 = .*073, Cohen's *d =* .56; Table 2; Fig. 2B).

**Table 2:** Digit span scores for the three groups

Language	Hebrew-speaking controls	Hebrew-speaking IDDs	English-speaking controls
English	9.0 (2.0)	7.9 (1.8)	10.7 (2.4)
Hebrew	10.5 (1.9)	8.0 (1.5)	-

Scores (mean (SD)) of Digit Span forward, in Hebrew and English. The score for each sequence was either zero or one: zero if there was at least one mistake/missing digit, and one if the recall was perfect. The total score for each participant for each condition is the number of the correctly reproduced sequences

*IDD* individuals with developmental dyslexia

Although the absolute average score of IDDs did not differ between Hebrew and English, this probably reflects an effectively larger span in Hebrew, since Hebrew digit-words are longer than English digit-words (i.e., one monosyllabic and eight disyllabic digit-words in Hebrew vs. one disyllabic and eight monosyllabic in English) and given the strong evidence that spans are shorter for longer words (Guérard et al., 2012; Guitard et al., 2018; Naveh-Benjamin & Ayres, 1986; Service, 1998).

Given IDDs' smaller gains when performing the DST in their native language, they seem to rely less on the advantage of vast exposure as compared to the general population (Jaffe-Dax et al., 2015; Lieder et al., 2019), and thus perhaps use a similar set of cognitive mechanisms for both native and non-native languages. We therefore hypothesized that their native- and non-native-language scores would be highly correlated. By contrast, controls gain substantially from long-term item-specific exposure. We hypothesized that for controls, the efficient utilization of exposure underlies their elevated scores in their native language, and that there would therefore be a reduced correlation between their first- and second-language scores. Figure 2C illustrates that this was indeed the case: for IDDs the correlation between DST scores in the two languages was highly significant (Pearson: *r =* .60, *p <* .001, Spearman *r =* .61, *p <* .001), whereas for controls it was not (Pearson: *r =* .25, *p =* .179, Spearman *r =* .27, *p =* .151; though the group difference between the strength of the correlations was only marginally significant: Fishers Z-test, *z =* 1.64, one-sided *p <* .05; Fig. 2C; Lenhard & Lenhard, 2014).

## Discussion

We administered the DST in native and second language, Hebrew and English respectively, and found that controls benefited more than IDDs from the use of native-language digit-words, supporting our hypothesis about reduced benefit from exposure/item frequency among IDDs. An additional support for the different effect of items’ frequency on the two groups was the lack of significant correlation between the DST scores in native and second language in the control group versus a high correlation among IDDs. This suggests an exposure-related contribution that affects controls’ spans, but not IDDs’ spans. We conclude that the reduced impact of item frequency in dyslexia could, to a large extent, explain the numerous reports on lower DST scores among IDDs.

The English-speaking control group that took part in the experiment allowed us to verify our assumptions on exposure: English DST of Hebrew-speaking controls was smaller than that of native English speakers, indicating that Hebrew-speaking controls' exposure to English has not been sufficient for reaching asymptotic performance, even though English is heavily used in Israel. Thus, in Fig. 1, for Hebrew speakers, English digit-words are on the left-hand side of the graph, and Hebrew digit-words are on the right-hand side.

Beyond the group × frequency interaction, the overall group difference is a replication of numerous previous reports on reduced STM in dyslexia across the board, in adults and in children with dyslexia (Gabay et al., 2012, 2015; Gabay & Holt, 2015; Hatcher et al., 2002; Henderson & Warmington, 2017; Jeffries & Everatt, 2004; Ramus & Szenkovits, 2008; Steinbrink & Klatte, 2008; Witton et al., 1998). The effect of item frequency, implemented by the usage of native versus second language in this study, is consistent with the extensive corpus of research on the effect of frequency on span scores (Hulme et al., 1997; Nimmo & Roodenrys, 2002; Roodenrys et al., 2002; Tremblay et al., 2016).

### Dyslexia: A learning difficulty

Our findings that IDDs benefit less from the use of native language digit-words are not consistent with an assumption that the core deficit in dyslexia is a fixed deficit with phonology or an increased internal noise, and indeed, the link between noise exclusion and reading abilities in dyslexia was shown to be weak (Nittrouer, 2012). If the deficit was fixed and not learning-centered, we would not expect to find a bigger relative difficulty for IDDs in the frequent condition since repetition could potentially compensate for noisier representations, and is not expected to increase group difference. In other words, theories that do not take into account the atypicality of the learning process, predict that group difference should not increase with exposure.

The results are consistent with theories focused on the atypicality of the learning process (Lum et al., 2013; Ullman, 2004), such as the anchoring deficit hypothesis (Ahissar, 2007; Ahissar et al., 2006), as illustrated in Fig. 1. In fact, according to any theory that is focused on learning rather than on reduced skills as a fixed characteristic of dyslexia (Nicolson et al., 2010; Nicolson & Fawcett, 2007), a group difference that increases with practice is theoretically predicted.

Another prediction derived from a learning-based approach is that IDDs will also underuse morphological representations, which heavily rely on linguistic structural regularities that are implicitly acquired over time due to the mass exposure to them. Indeed, it had been previously reported that when compared with the general population, IDDs rely less on morphology when acquiring new vocabulary (Kimel & Ahissar, 2019), they have reduced sensitivity to morphological similarity between words (Schiff & Ravid, 2007), they exhibit impaired morphological relationship judgments (Ben-Dror et al., 1995), and they benefit less from morphology when reading (Kimel & Ahissar, 2019; though see Bitan et al., 2020).

Further support for a less usable long-term knowledge among IDDs can be derived from studies on consonant distribution in spoken native language. Artuso and colleagues (Artuso et al., 2021) tested 45 children with dyslexia and 45 children without dyslexia, all of whom were native Italian speakers. In the experiment, a triplet of letters was presented on each trial. The first two letters were either a high-frequency pair or a low-frequency pair in spoken Italian. The link with the third letter was always linguistically impossible (e.g., in the triplet *CHB,* CH is a high-frequency pair, and the link between H and B is impossible in Italian). Participants were requested to actively maintain the triplet in memory. Then, they were requested to substitute one letter in the triplet, and to actively maintain the new triplet in memory. At the end of the trial participants were presented with a single letter and asked to indicate whether this letter was part of the most recently studied triplet or not. The letters for which the participants were expected to provide a negative answer were either lures (i.e., the letter in the initial triplet, before substitution) or letters that were not presented in that trial at all (Artuso & Palladino, 2018). Control participants were slower in rejecting a lure consonant letter when it was part of a high-frequency pair of consonants as compared to when this letter was part of a low-frequency pair. But this was not the case for IDDs, for whom the frequency manipulation had no significant effect, suggesting, in line with a reduced benefit of exposure, that long-term information of consonant pair distribution had a weaker (if any) effect on their STM (Artuso et al., 2021).

Given the evidence on long-term knowledge effects on performance in STM tasks, and the reports on reduced benefits from input statistics in dyslexia, Kimel and colleagues administered a series of span experiments, with frequent and infrequent syllables. They found that both IDDs and controls’ spans were higher for frequent syllables, but IDDs benefited less than controls from syllable frequency, suggesting that they benefit less than controls from each exposure to a syllable in everyday language (Kimel et al., 2020, 2022). Thus, although massive exposure provides an opportunity to improve and sharpen phonological representations, it cannot compensate for any atypical learning dynamics with a lower benefit from each exposure.

### STM, word frequency, and reading proficiency

STM deficits are likely to contribute to reduced reading proficiency (Jacobson et al., 2011; Jeffries & Everatt, 2004; Naveh-Benjamin & Ayres, 1986; Nicolson, 1981; Roodenrys & Stokes, 2001; Snowling et al., 1986; Snowling, 1981), and reading proficiency affects STM since literacy and writing acquisition also sharpen phonological and lexical representations, which in turn boost STM performance (Demoulin & Kolinsky, 2016). IDDs’ exposure to print is reduced, and this could have a potential impact on their performance in span tasks, reducing their measured memory span. Using digit-words, which are very frequent in spoken language, is advantageous since unlike other words, exposure to digit-words does not strongly rely on reading (Dehaene & Mehler, 1992). Still, in studies with literate adults we cannot completely rule out some contribution of their reading practice.

Reduced utilization of item frequency is expected to impede reading proficiency, which heavily relies on benefits from familiarity with words (e.g., Carlisle & Katz, 2006), and syllables (e.g., Ashby & Rayner, 2004). Interestingly, results of previous reading studies potentially suggest that IDDs’ reading benefits adequately from word frequency (Davies et al., 2007; Van der Leij & Van Daal, 1999). We interpret this result as a consequence of a confound: words carry meaning, and thus frequent words provide a semantic advantage, which we do not expect to be smaller for IDDs.

### Effect of semantics on performance of IDDs in STM tasks

In contrast to previous studies that have examined the effect of word frequency on performance in span tasks, our study is unique in its full control for semantics and all that entails. Semantics is utilized by participants in span tasks (Hulme et al., 1991): Word pleasantness (Monnier & Syssau, 2008), word concreteness (Allen & Hulme, 2006; Romani et al., 2008; Walker & Hulme, 1999), word imageability (Bourassa & Besner, 1994), and the semantic similarity between the words in the series to be remembered (Saint-Aubin et al., 2005) - all have an effect on the performance in span tasks, but these factors are often not controlled for when comparing frequent and infrequent word spans, including in studies with IDDs.

Several studies have observed a larger group difference in span task performance between IDDs and controls when using infrequent words as opposed to frequent ones (Kibby, 2009a, b; Snowling et al., 1986), and these appear to be at odds with the findings of the present study. However, these differences can be accounted for by the use of semantics, and this line of thought is especially convincing since IDDs are considered to have intact semantics (Bishop & Snowling, 2004; Kibby, 2009a), and were even proposed to rely more heavily on semantic processing as compared to controls, potentially using semantics as a compensatory mechanism (Kibby, 2009b; Klimovich-Gray et al., 2023), in line with studies showing more reliance on an orthography-semantics mapping as compared to an orthography-phonology mapping in less proficient readers (Brice et al., 2022; Siegelman et al., 2020).

Furthermore, the co-occurrence of word frequency and availability of semantic memory strategies is especially true in studies with children, when the low-frequency condition comprises words that the participants don’t know yet (Kibby, 2009b), and thus these are effectively nonwords for them, with semantic strategies becoming largely impossible to implement. Such designs are prone to measure even bigger control-IDD group differences for infrequent words, since controls, who have more exposure to written materials than IDDs, are likely to have richer vocabularies (Casalis et al., 2004; Lyon et al., 2003), and thus they will potentially be able to make more use of semantics, resulting in an additional relative disadvantage for IDDs in the infrequent condition.

Therefore, in this study, we derived a way to dissociate semantic frequency from phonological frequency, by using the DST conducted in two languages, and thus semantics was equated by design.

### Hebrew versus English: Beyond exposure and semantics

Hebrew and English digit-words differ across several dimensions, and one of them is the average number of syllables: in Hebrew one digit-word in monosyllabic and the rest are disyllabic, and English displays the opposite pattern. Thus, Hebrew digit-words are longer than English digit-words, making the Hebrew DST harder (Guérard et al., 2012; Guitard et al., 2018; Naveh-Benjamin & Ayres, 1986; Service, 1998). One could then ask whether the longer digit-words in Hebrew present a greater challenge to IDDs’ memory, resulting in a larger group difference in the native language condition. Kibby (2009a, b) tested the effect of word length on spans with children with and without dyslexia, and found that word length did not have a stronger effect on IDDs’ performance in span tasks as compared to controls (Kibby, 2009a, b; see also Steinbrink & Klatte, 2008), thus making digit-word length less likely to explain our results.

Moreover, if English digit-words were less demanding for IDDs due to their shorter length, we would expect a reduced relative difficulty for native English-speaking IDDs when tested with DST in English; however, this is not the case. Performance in the DST has been assessed many times for English-speaking IDDs and controls, and the reported group differences were similar to those found for the Hebrew speakers in our study, both for the scaled score (Hatcher et al., 2002; Witton et al., 1998) and for forward score alone (Hatcher et al., 2002; Richardson et al., 2011). This cross-language similarity in IDDs’ difficulty further supports the long-term frequency/exposure hypothesis over the fixed phonological deficit hypothesis: Although phonological complexity differs between languages, the prevalence of digit-words for native speakers is expected to be roughly similar, resulting in a similar group difference between controls and IDDs. Longer word length likely hampers the performance of both groups; however, controls are able to compensate for this decline through their extensive familiarity with digit-words in their native language, whereas IDDs are less able to.

Another potential confound is phonological neighborhood size, which was shown to affect auditory span performance, with sequences of words with more phonological neighbors remembered better than sequences of words with a smaller number of neighbors (Allen & Hulme, 2006; Roodenrys et al., 2002; and there are similar findings for memory of written words and orthographic neighbors – Jalbert et al., 2011a, b). While it is hard to directly compare neighborhood sizes between Hebrew and English due to Hebrew processing heavily relying on root-based morphology (Frost et al., 1997, 2000; Velan et al., 2005), it is unlikely that neighborhood size could be the underlying cause for a larger group difference in our study since IDDs were shown to have adequate benefits from phonological neighborhood size in span tasks (Thomson et al., 2005) and other memory tasks (Mainela-Arnold et al., 2010).

### Memory for item identity versus memory for order

STM for item order and STM for item identity seem to rely on two separate mechanisms (Attout et al., 2012; Brown et al., 1999; Gupta, 2003; Henson, 1998; Hurlstone et al., 2014; Nairne & Kelley, 2004), and a number of studies have reported a specific deficit with STM for order in dyslexia (Duyck et al., 2014; Giorgetti & Lorusso, 2019; Leclercq & Majerus, 2010; Perez et al., 2012; Peter et al., 2018; Romani et al., 2015; Schraeyen et al., 2019; though see Litt & Nation, 2014; Majerus & Cowan, 2016).

The DST does not allow us to directly distinguish between memory for item and memory for order, but previous literature suggests that STM for order is less affected by long-term representations as compared to STM for item identity (Fallon et al., 1999; Majerus et al., 2007; Majerus & D’Argembeau, 2011; Nairne & Kelley, 2004; Perez et al., 2012; Saint-Aubin & Poirier, 1999). In the current study, the difference in long-term representation is central to the shift from second language to native language, and therefore the group × language interaction reported in this study is probably more related to memory for item identity than to memory for order (Litt & Nation, 2014), although a reduced memory for order among IDDs can still contribute to the overall group difference.

### The effect of long-term knowledge on STM in adults versus children with dyslexia

Overall, we predict a larger group difference between IDDs and controls in adults compared to children in the DST conducted in their native language, due to adults' longer exposure to their everyday native language. And indeed, examining studies that assessed differences in DST in adults with and without dyslexia, and in children with and without dyslexia, it seems that it might be the case (Gabay et al., 2015; Gabay & Holt, 2015; Hatcher et al., 2002; Henderson & Warmington, 2017; Howes et al., 1999; Jeffries & Everatt, 2004; Nelson & Warrington, 1980). The average effect size of the group difference in DST for adults (mean age range 20–25 years, across five studies, including the current study) was found to be 1.42 (range 1.12–1.67), whereas for children (mean age range 9–11.5 across three studies), it was found to be 1.21 (range .81–1.46). The relative performance of IDDs might be getting worse with age due to IDDs’ reduced benefit of each exposure, but a more systematic study, and especially studies testing group × frequency interaction without the confound of semantics, are needed to robustly answer the question of dynamics with age.

Group differences may also be task-dependent (Henderson & Warmington, 2017; Kimel & Ahissar, 2019) and be heavily influenced by stimuli properties. For example, in a lexical decision task conducted with students with and without dyslexia in the second and fourth grades (Luque et al., 2013), group difference for correct word recognition did not change between the two age groups for infrequent words, whereas for frequent words, group difference got smaller with age, suggesting an emergent semantic effect. Potentially, this could also be explained by greater exposure leading to a compensation of the fixed phonological deficit in IDD. However, this doesn’t seem to be the case, as syllable frequency, manipulated in the same study in a 2 × 2 design with word frequency, could not account for these effects, and thus, this reduction in the group difference with age was more likely to stem from semantics rather than phonology. On the contrary, in a study testing spans of frequent words, infrequent words, and non-words in 9-year-old children with and without dyslexia, and in poor readers with general learning difficulties, no significant group differences were found between controls and IDDs (McDougall & Donohoe, 2002). Therefore, while amount of exposure and age are central to the performance level and to the magnitude of the group difference, task properties and stimuli characteristics are of great importance as well.

## Summary

The significant difference in Digit Span performance between controls and IDDs has been previously interpreted as reflecting generally limited verbal/phonological STM in dyslexia. We now suggest that this group difference largely stems from IDDs’ reduced benefit of exposure, specifically hampering IDDs’ relative performance in the DST since digit-words have a high frequency in spoken everyday language.

We tested this hypothesis in adults with and without dyslexia by conducting a Digit Span experiment in native language and in second language, thus manipulating item frequency without introducing a difference in semantics. Controls had a higher native-language benefit as compared to IDDs, suggesting that IDDs’ poor Digit Span, at least partially, reflects impaired accumulative long-term learning. Considering the well-established body of research indicating the significant impact of long-term memory on performance in STM tasks, it is important to interpret the underlying causes of performance levels in such tasks in the typical and in neuroatypical populations with caution.

## Data Availability

Upon request.

## Code availability

Analysis code, upon request.
